# Current Knowledge about ActiGraph GT9X Link Activity Monitor Accuracy and Validity in Measuring Steps and Energy Expenditure: A Systematic Review

**DOI:** 10.3390/s24030825

**Published:** 2024-01-26

**Authors:** Quentin Suau, Edoardo Bianchini, Alexandre Bellier, Matthias Chardon, Tracy Milane, Clint Hansen, Nicolas Vuillerme

**Affiliations:** 1 AGEIS, Université Grenoble Alpes, 38000 Grenoble, France; quentin.suau@univ-grenoble-alpes.fr (Q.S.); abellier@chu-grenoble.fr (A.B.); matthias.chardon@gmail.com (M.C.); tracymilane@gmail.com (T.M.); c.hansen@neurologie.uni-kiel.de (C.H.); 2 Department of Neuroscience, Mental Health and Sensory Organs (NESMOS), Sapienza University of Rome, 00189 Rome, Italy; 3 CHU Grenoble Alpes, Université Grenoble Alpes, Inserm CIC 1406, 38000 Grenoble, France; 4 UNESP Human Movement Research Laboratory (MOVI-LAB), Department of Physical Education, Bauru Sao Paulo State University, Bauru 17033-360, SP, Brazil; 5 Department of Neurology, Kiel University, 24105 Kiel, Germany; 6 LabCom Telecom4Health, Orange Labs & Université Grenoble Alpes, CNRS, Inria, Grenoble INP-UGA, 38000 Grenoble, France; 7 Institut Universitaire de France, 75005 Paris, France

**Keywords:** ActiGraph GT9X Link, accuracy, precision, step, energy expenditure, wearable devices, eHealth, digital health, mobile health, mHealth, systematic review

## Abstract

Over recent decades, wearable inertial sensors have become popular means to quantify physical activity and mobility. However, research assessing measurement accuracy and precision is required, especially before using device-based measures as outcomes in trials. The GT9X Link is a recent activity monitor available from ActiGraph, recognized as a “gold standard” and previously used as a criterion measure to assess the validity of various consumer-based activity monitors. However, the validity of the ActiGraph GT9X Link is not fully elucidated. A systematic review was undertaken to synthesize the current evidence for the criterion validity of the ActiGraph GT9X Link in measuring steps and energy expenditure. This review followed the PRISMA guidelines and eight studies were included with a combined sample size of 558 participants. We found that (1) the ActiGraph GT9X Link generally underestimates steps; (2) the validity and accuracy of the device in measuring steps seem to be influenced by gait speed, device placement, filtering process, and monitoring conditions; and (3) there is a lack of evidence regarding the accuracy of step counting in free-living conditions and regarding energy expenditure estimation. Given the limited number of included studies and their heterogeneity, the present review emphasizes the need for further validation studies of the ActiGraph GT9X Link in various populations and in both controlled and free-living settings.

## 1. Introduction

It is now recognized that regular exercise is a cost-effective method to maintain a good health status and to decrease the risk of chronic disease [1,2]. Walking is a simple and low-cost activity that can be integrated easily into daily life activities [3]. The number of steps per day is a marker of physical activity and is related to several conditions such as cardiovascular disease [4], dementia [5], cancer [4], and overall mortality [6,7,8,9]. The World Health Organization has proposed guidelines to promote a sufficient level of physical activity to maintain or improve health status [10]. To this end, one of the most popular messages is to achieve 10,000 steps per day to optimally influence health status [11] and this threshold was confirmed in a recent large study [4]. 

Accelerometry is a common tool to assess numbers of steps per day [12] and a growing number of wearable devices, either consumer- or research-grade, are available to quantify physical activity and sedentary behavior through step count. The spread of these wearable physical activity trackers could also encourage individuals to increase their levels of physical activity through self-monitoring [13,14]. However, implementation in clinical practice requires that feedback provided to the users is accurate and reliable. ActiGraph devices (ActiGraph Corp, Pensacola, FL, USA) are among the most common research-grade accelerometers used in research [15,16]. The GT9X Link is a recently developed activity monitor available from ActiGraph that has been used to assess the validity of various consumer-based activity monitors under free-living conditions [17,18]. The ActiGraph GT9X is a small wearable device integrating an inertial measurement unit (IMU) composed of a triaxial gyroscope, a triaxial magnetometer, and a secondary triaxial accelerometer. It can be worn for long periods (14 days, depending on enabled option) in different positions on the body (e.g., wrist, ankle, or hip), using manufacturer accessories. Moreover, GTX9 can provide information to the user, such as real-time feedback on steps and energy expenditure achieved during the day. The device is coupled with dedicated software (ActiLife) used to initialize the device prior to the monitoring period, and to download the data after the test. Signals can be processed using a normal filter (NF) or a low-frequency bandpass filter (LFE), which increase sensitivity to capture lower-intensity activities. After signal processing, physical activity energy expenditure (not including basal metabolic rate) can be computed using five algorithm options available to users. Steps can be computed using three different methods from ActiGraph GT9X data: (1) directly from information displayed on the sensor screen, also called moving average vector magnitude (MAVM) [19,20]; (2) after postprocessing with NF; and (3) after postprocessing with LFE. Eleven measures of kcal can be obtained from an ActiGraph GT9X device: (1) a single measure from information displayed on the sensor screen (MAVM); (2) five measures from NF; and (3) five measures from LFE.

A recent review reported the results of 21 published articles that have investigated the criterion validity of ActiGraph devices for step counting and distance estimation in healthy adults and older adults [16]. Interestingly, the authors concluded that no study on the criterion validity of the ActiGraph GT9X was available [16]. 

Since the ActiGraph GT9X Link has been used in previous publications to monitor energy expenditure and step count, but no study has examined the current knowledge on its criterion validity, the aim of this systematic review is to fill this gap in the literature by summarizing the current state of evidence on this topic. The results of this systematic review will inform researchers, clinicians, and consumers on the criterion validity of ActiGraph GT9X Link device for estimating steps and energy expenditure across the age span, in various populations, in both controlled and free-living settings. Moreover, this review will help address potential unmet needs in this regard.

## 2. Materials and Methods

The review protocol of the present systematic review was registered within the International Prospective Register of Systematic Reviews (PROSPERO) (registration number: CRD#42023418081) in April 2023. It was developed based on the Preferred Reporting Items for Systematic Reviews and Meta-Analyses (PRISMA) [21] and the Cochrane Handbook for Systematic Reviews guidelines [22]. Since this systematic review is limited to publicly available materials, it did not require any ethical approval.

### 2.1. Eligibility Criteria

Inclusion criteria: Studies were included if they (1) were original articles published in English-language peer-reviewed journals, (2) included human participants with no restrictions on age, gender, health status, or type or stage of disease, and (3) simultaneously reported outcome data from the ActiGraph GT9X Link activity monitor (steps or energy expenditure) and a valid criterion measure. As in a recent systematic review [23], only direct observations (video recorded or not) were considered valid criterion measures for steps, and doubly labelled water or direct and indirect calorimetry as the only valid criterion measures for energy expenditure.

Exclusion criteria: Studies were excluded if they (1) were case reports, abstracts, editorials, letters to the editor, case studies, books, chapters, reviews, meta-analyses, or other gray literature materials (i.e., government reports, policy statements, issues papers, conference proceedings, preprinted articles, theses, and dissertations); or (2) did not employ an Actigraph GT9X Link activity monitor to measure steps or energy expenditure; or (3) did not use valid criterion measures of steps or energy expenditure [23]; or (4) involved fewer than 10 participants [24,25].

### 2.2. Data Sources and Search Strategy

Three databases, PubMed, Web of Science, and SPORTDiscus, were searched systematically to identify studies satisfying the search criteria. A first search was conducted in April 2023, and this search was repeated in December 2023 before the final review.

The search focused on keywords related to three concepts, namely, (1) the activity monitor (“GT9X”), (2) variations on the terms (Validity/Validation (valid*) OR Accuracy (accura*) OR Comparison/Comparative (compar*) OR Equivalence (equival*) OR Agreement), and (3) outcomes (step*, stride*, “energy expenditure”). Keyword categories were combined as follows: (1) AND (2) AND (3).

### 2.3. Study Selection

Two independent reviewers (QS and TM) screened the titles, abstracts, and keywords of all the studies found in the search to identify potentially relevant articles. Duplicates were manually removed. The same two reviewers then screened full-length text articles to assess their eligibility according to inclusion and exclusion criteria. In case of discrepancies or disagreements and if subsequent discussions between the two reviewers were inconclusive, a third review team member (MC) was contacted to arbitrate until a consensus was found. 

### 2.4. Data Extraction

First, a data extraction form was created and validated by the team members. Data extraction was then performed independently by 2 reviewers (QS and TM) who were not blinded to the authors or journals. 

The following 6 groups of data were extracted from each article retrieved: (1) the study characteristics, (2) the sample description; (3) the outcomes examined (i.e., measures of steps and/or energy expenditure); (4) the protocols used to assess the validity of the Actigraph GT9X Link (study setting, activity type, criterion measure); (5) criterion validity indices (e.g., mean average percentage of error (MAPE), mean percentage of error (MPE), etc.); and (6) main results obtained.

Metrics such as MPE were extracted directly from selected studies when available, or computed using other reported statistics (i.e., group mean) to allow comparison across studies as in recent systematic reviews [24,25], using the following equation: (GT9X—Criterion)/Criterion, where GT9X is the group mean value (step, kcal, or meters) provided by the GT9X activity monitor, and Criterion is the group mean value (step, kcal, or meters) provided by the criterion measure.

Details from each independent reviewer (QS and TM) were compared. Any disagreement or inconsistency between the two reviewers was resolved by consensus or discussion with a third review team member (MC)

### 2.5. Methodological Quality

As in two recent systematic reviews [26,27], the risk of bias was calculated and the quality assessment was performed using a modified version of the Hagströmer Bowles Physical Activity/Sedentary Behavior Questionnaire Checklist (HBQC) [28] (see Supplementary Table S1). HBQC is a modified version of the Downs and Black [29] checklist specific to physical activity assessments. The 3 questions (5, 14, 19) that did not apply to comparisons of objective measures were removed from the original 22-item checklist, for a remaining total of 19 items. This modified version of the HBQC was extracted from recent reviews [26,27] and is presented in Supplementary Table S1.

Quality assessments were performed independently by 2 reviewers (QS and TM) who were not blinded to the authors or journals. In case of discrepancies or disagreements between the two reviewers regarding their quality assessment decisions and if subsequent discussions were inconclusive, a third review team member (MC) was contacted to arbitrate until a consensus was found. 

### 2.6. Data Synthesis

Given the limited number of included studies and their heterogeneity, we were unable to conduct meta-analyses of the extracted data and only a qualitative synthesis of data was performed. Measurement accuracy focused on acceptable limits of percentage difference of ±3% in controlled settings (i.e., laboratory and semi-free-living settings) and percentage difference of ±10% in free-living settings [30,31]. Correlation coefficients were interpreted as follows: 0 to <0.2, very weak; ≥0.2 to <0.4, weak; ≥0.4 to <0.6, moderate; ≥0.6 to <0.8, strong; and ≥0.8 to 1.0, very strong [32].

## 3. Results

### 3.1. Study Selection

**Figure 1:**
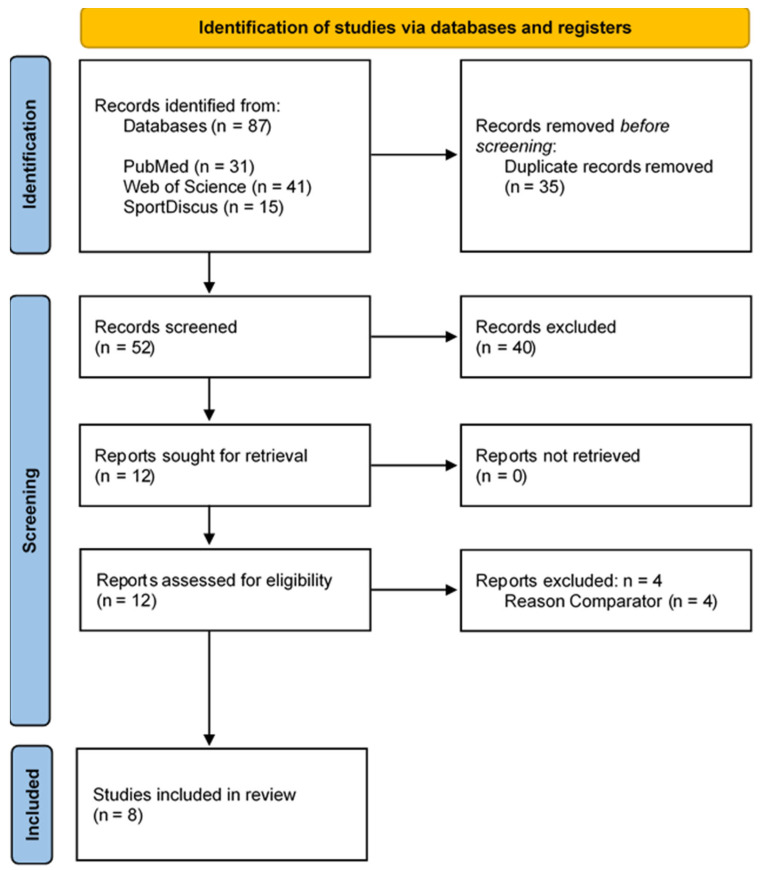
Flow diagram of the articles included in the review. The numbers of original articles (i.e., not duplicates) are indicated at each stage of the search.

The electronic searches of the three electronic databases (PubMed, Web of Science, and SPORTDiscus) resulted in a total of 87 records. After removing duplicates (n = 35), 52 records remained. After screening titles, abstracts, and keywords, 12 full texts were read to verify and confirm their eligibility. After full-text screening, four studies were excluded and eight studies fulfilled the eligibility criteria and were included in this systematic review [19,33,34,35,36,37,38,39]. The study selection process is illustrated in Figure 1.

**Figure 2:**
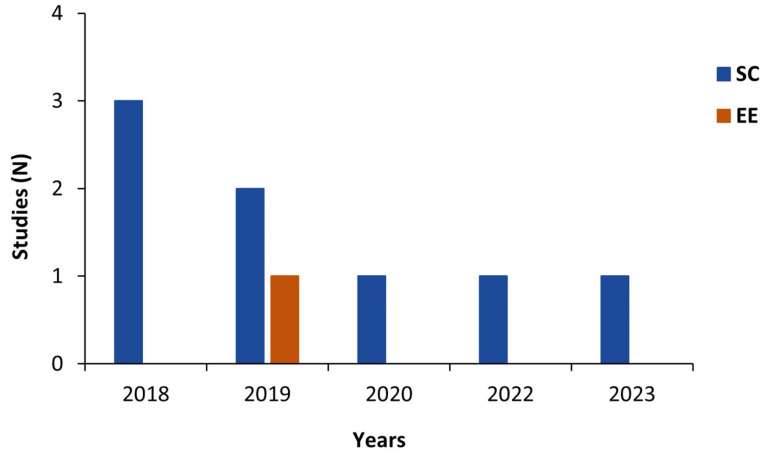
Number of studies published per year by measurement type. EE: energy expenditure; SC: step count.

**Figure 3:**
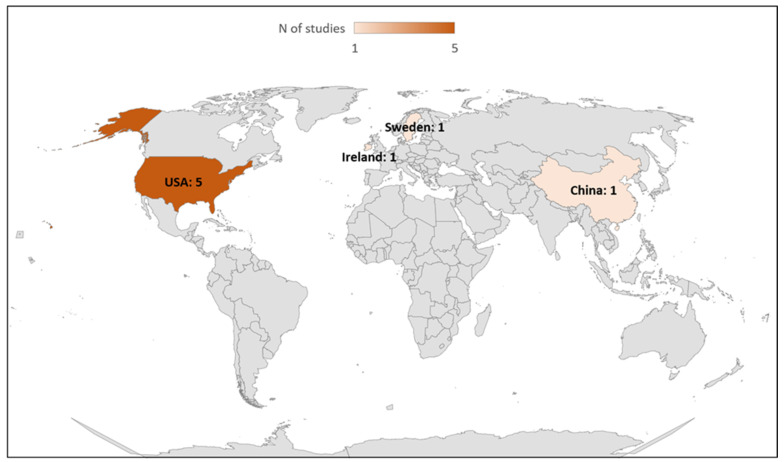
Number of studies published by country.

Among these eight included studies, seven studies evaluated step counting [19,33,34,36,37,38,39] and one study (12.5%) evaluated the energy expenditure measurement [35] provided by the ActiGraph GT9X. The general characteristics of the studies included are summarized in Table 1 and Table 2. Numbers of studies published per year and by country are shown in Figure 2 and Figure 3, respectively.

### 3.2. Participant Characteristics

Sample size: The eight included studies combined a sample size of 558 participants (414 healthy individuals and 144 individuals with pathological conditions). The mean sample size was 69 ± 86 participants, ranging from 12 [19] to 258 [38]. 

Sex: All studies (n = 8, 100%) included both female and male participants, totaling 249 females (44.6%) and 309 males (55.4%).

Age: In the eight studies, the 558 participants were 49.4 ± 20.1 years old: 218 young adults ranging from 21.4 ± 1.1 [37] to 35 ± 13 years old [19] (n = 5, 62.5%) [19,35,36,37,38], 110 middle-aged adults ranging from 49.2 ± 14.0 years old [33] to 50.2 ± 5.9 years old [38] (n = 2; 25%), and 230 older adults ranging from 69 ± 3.2 [39] to 72.6 ± 6.9 [38] (n = 3; 25%) [34,38,39]. 

Health status: All studies included a homogenous population in terms of health status. Six studies (75%) included 414 healthy participants and two (25%) included 144 individuals with pathologies, namely 30 patients with multiple sclerosis (n = 1, 12.5%) [33] and 114 patients with peripheral artery disease (n = 1, 12.5%) [34]. Table 3 presents the participant characteristics of the eight studies included. 

In the following two sections, we will discuss the study features and main findings for criterion validity of ActiGraph GT9X for step counting and energy expenditure. 

### 3.3. Studies Assessing Validity of ActiGraph GT9X Link for Step Counting

**Figure 4:**
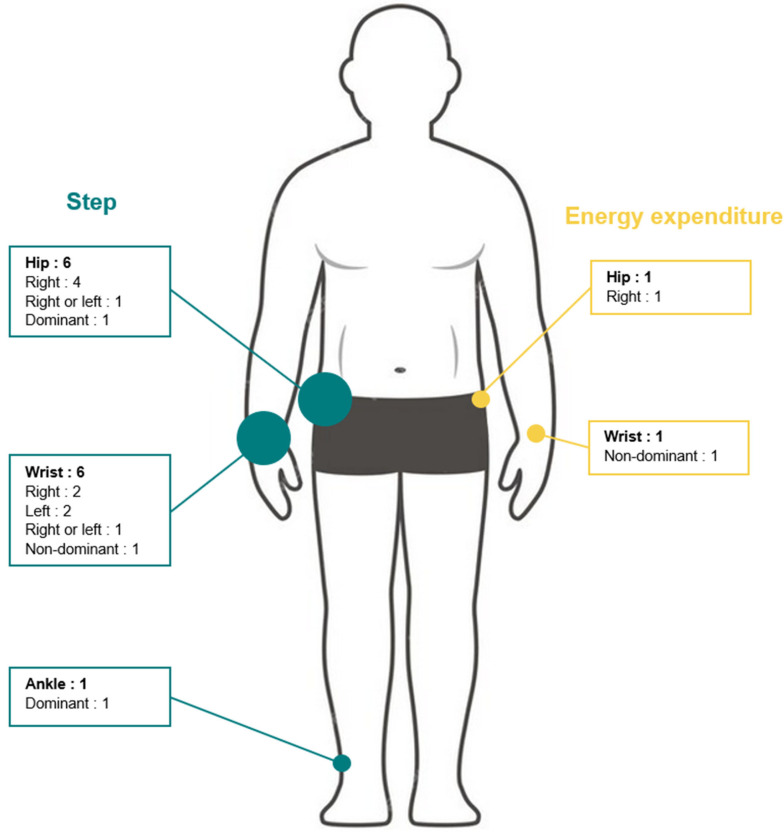
Numbers and placement of ActiGraph GT9X devices in included studies.

Device positioning: The positioning of GT9X devices differed across studies: hip (n = 6) [19,33,34,36,38,39], wrist (n = 4) [19,36,37,38], and ankle (n = 1) [39]. Among them, two studies positioned monitors on one hip and on one wrist [36,38], two on one hip [33,34], one on two wrists [37], one on one hip and on two wrists [19], one on one wrist and one ankle [39]. Only one study reported whether the device was placed on the dominant or non-dominant side (n = 1) [39], five studies reported the placement side (i.e., right or left) [19,33,34,36,37], and two studies positioned devices on both sides [35,38]. A schematic illustration of device placement is depicted in Figure 4. 

Details regarding ActiGraph GT9X setups including device placement, sample frequency, extraction methods, and activation/deactivation of IMU option are reported in Table 4.

Criterion measure: As criterion measures for step count, three studies used video recorded with at least two observers [19,33,37], two studies used direct observation with a single observer during the task [34,36] and two studies used direct observation or/and video recording [38,39].

Validity indices: To investigate the association between criterion and GT9X for step count, one study used Pearson’s correlation coefficient [19], one study used intra-class correlation (ICC) [39], one study used Spearman’s correlation coefficient [33], one study used root mean square of error (RMSE) [39], and one study used coefficient of variation (CV) [38]. 

Error measures: To quantify difference between measurement tools, three studies reported mean absolute percentage of error (MAPE) [19,38,39] and one study median absolute percentage of error (MeAPE) [33]. Four studies reported mean percentage of error (MPE) [34,37,38], three studies reported mean difference (MD) [19,36,37], one study computed MAD (median absolute deviation) [39], one study used mean absolute error MAE [39], two studies illustrated results in Bland and Altmann plots [33,34], and one study calculated the percentage of manually counted steps detected by the device [19]. Significative difference between criterion and GT9X measurement was tested in one study using Student’s *t*-test [19], in one study using the Wilcoxon test [33], and in one study without the applied test [36]. As was carried out in recent systematic reviews [24,25], we further computed MPE for the two other studies using group means [33,36].

Experimental settings: Among the studies that investigated GT9X step-counting accuracy, six studies (86%) used laboratory settings [33,34,36,37,38,39], one (14%) a semi-free-living setting [39], and one (14%) free-living conditions [19]. The duration of acquisition ranged from 1 to 6 min, with two studies (29%) specifying 1 min [36,37], one study (14%) 3 min [39], two studies (29%) 5 min [33,38], one study (14%) 6 min [34], and one study (14%) 1 day (at least 14 h) [19]. The six studies conducted in laboratory settings included treadmill walking or running (n = 2, 33%) [36,38], overground single-task walking (n = 2, 33%) [33,34], and both treadmill and overground dual-task walking (n = 2, 33%) [37,39]. Table 5 describes the experimental settings of the included studies. Supplementary Table S2 shows the results of studies examining ActiGraph GT9X step-counting validity associated with walking speed, when these data were available.

#### Main Findings on Step Count

Here, we present the main findings regarding the step-count validity of the GT9X. For the sake of clarity, studies conducted on treadmill walking, overground walking, semi-free-living settings, and free-living conditions are presented separately. 

Treadmill walking: Using treadmill walking, three studies [36,38,39] investigated the step-counting accuracy of the GT9X device across several speeds (ranging from 0.28 to 3.14 m/s). Across 86 comparisons, 94% underestimated steps (MPE ranged from −98.31% to 1.26%) counted by the criterion measure regardless of the wearing position, walking speed, and population. Of these, 13 (15%) were within ±3% measurement error and 73 (85%) were below −3% measurement error. 

One study [37] conducted on treadmill walking investigated the step-counting accuracy of GT9X devices placed on both wrists for subjects walking at a single speed (4.8 km/h) while holding handrails. The authors found MPE values of −84.7 ± 27.2% and −89.1 ± 21.1% for the GT9X devices placed at the right and left wrist, respectively.

Overground walking: For overground walking, participants performed level-ground walking [33,34] with self-selected speed instructions including as fast as possible (i.e., ATS 6MWT guidelines) [34], comfortable, slow, and fast self-selected speeds [33], complex walking (i.e., up and down stairs) [39], dual-task walking (i.e., while carrying a box and with a rollator) [39], and fixed-handed tasks (i.e., with a stroller, with a baby doll, with grocery bags) [37]. 

Across walking speeds and filtering processes, these studies [33,34] reported a general underestimation (−46% to 0%) of steps counted by the criterion measure. Anens and collaborators [33] reported a lower MeAPE value with LFE (1.9%) than normal filter (23.1%) or MAVM (16.9%), regardless of walking speeds. Of these 10 comparisons, 1 (10%) was within ±3% measurement error and 9 (90%) were below −3% measurement error.

In the context of overground dual-task walking, two studies [37,39] conducted experiments with various household tasks and reported general underestimation (−94.3% to 0.4%) of step count by the criterion measure in all tasks regardless of the wearing position. Of these 16 comparisons, 4 (25%) were within ±3% measurement error and 12 (75%) were below −3% measurement error.

Semi-free-living settings: One study (14%) [39] evaluated GT9X monitors’ step measurement in 18 healthy older adults when placed on the dominant ankle and on the dominant hip in a semi-free-living task that involved dusting for 3 min. Tedesco and collaborators [39] found that when dusting, steps were underestimated by the GT9X monitors on the hip (MPE = −89.81 ± 14.23%) and the ankle (MPE = −90.34 ± 17.2%). This comparison was above 10% measurement error.

Free-living settings: Only one study (14%) [19] investigated the step-counting accuracy of GT9X devices positioned on the right hip and on both wrists of 12 heathy young adults and recorded steps according to MAVM, LFE, and normal filters in free-living settings. The authors found that recording with the LFE overestimated steps (128.1% to 219.7% of steps counted by the criterion measure) (*p* < 0.05) and with MAVM underestimated steps (69.9% to 91.0% of steps counted by the criterion measure), regardless of device positioning. The normal filter underestimated steps (69.2% of step counted by the criterion measure) when worn on the hip (*p* < 0.05) and overestimated them (109.0% to 122.2% of the criterion measure) when worn on the wrist. MPE was not reported or calculated from the group mean. Of these nine comparisons, one (11%) was within ±10% measurement error, three (33%) were below −10% measurement error, and five (56%) were above 10% measurement error.

### 3.4. Studies Assessing Validity of ActiGraph GT9X Link for Energy Expenditure

Only one study investigated this aspect [35]. Supplementary Table S3 shows the results of studies examining the validity of the ActiGraph GT9X in measuring energy expenditure associated with walking speed, when the data were available. 

Device positioning: In this study, the ActiGraph GT9X devices were placed simultaneously on the right hip and on the non-dominant wrist (Figure 2), sample frequency was set at 30 Hz, and no information regarding IMU option or applied filtering method was reported (see Table 4).

Criterion measure: The authors computed energy expenditure through indirect calorimetry (Vmax Encore 29 System; VIASYS Healthcare Inc, Yorba Linda, CA, USA) as a criterion measure using Freedson’s VM3 combination equation. No information regarding baseline conditions prior to the test were reported (e.g., fasting, time of day, exercise, etc.). 

Validity indices: The Pearson correlation coefficient and ICC were used to assess the criterion validity. 

Error measures: Difference between measurements was highlighted using MAPE. Significative difference between criterion and GT9X measurement was tested using one-way ANOVA with Games–Howell post hoc test results and effect size (Cohen’s d) reported.

Experimental settings: Energy-expenditure measurement accuracy was assessed in a laboratory setting. Participants were instructed to walk or run on a treadmill for 3 min at five speeds ranging from 4.80 to 11.28 km/h. This study was conducted in an indoor environment (see Table 5).

#### Main Findings on Energy Expenditure

Here, we present the main findings regarding the energy-expenditure validity of the GT9X. In the study by Ho and collaborators [35], energy-expenditure measurement accuracy was investigated across five speeds (0.28 to 3.14 m s^−1^) and two wearing positions (hip and wrist) in 90 healthy adults. The authors reported an underestimation of energy expenditure compared to the criterion. Regarding placement, the GT9X positioned on the hip provided more accurate energy-expenditure values (MAPE: 4.73 to 10.40%) than the one positioned on the wrist (MAPE: 21.43 to 63.86%). 

The ActiGraph GT9X device positioned on the hip demonstrated a homogeneous accuracy across walking speeds (range from ±4.73 to ±6.31%), except at 11.28 km/h when the accuracy was reduced. Conversely, the accuracy of the device placed on the wrist decreased with the increasing gait speed. MPE was not reported or calculated from the group values. Of the total 10 comparisons, all were below −3% measurement error.

**Figure 5:**
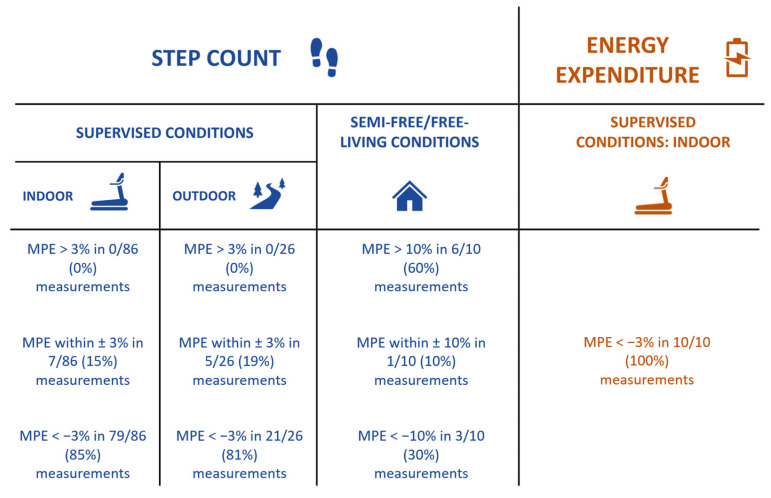
Accuracy of ActiGraph GT9X Link for step-count (blue) and energy-expenditure (orange) estimation across different experimental settings. MPE: mean percentage of error.

A summary of the accuracy of the ActiGraph GT9X for both step count and energy expenditure across different experimental settings is shown in Figure 5.

### 3.5. Methodological Quality

The total quality score ranged from 12 to 15 with an average of 12.9 ± 1.1. Individual study quality is indicated in Supplementary Table S4. The study quality assessment demonstrated homogeneous low external (1.0 ± 0.0 out of 3) and medium internal (5.0 ± 0.0 out of 7) validity scores, whereas study reporting was high in general (6.9 ± 1.1 out of 9). Seven studies (82.5%) [19,34,35,36,37,38,39] did not report the education level of participants, therefore the response to question 4 was considered as “no”. Previous studies [40,41] and a systematic review [16] have shown that data processing of ActiGraph devices influences steps or energy-expenditure outcomes. Therefore, we systematically answered no to question 6 for studies that did not report data processing for the GT9X (i.e., filtering options, sample frequencies, or epochs). 

No study reported an attempt to blind research staff to activity levels or participant characteristics (question 13) and reported a sample size (question 19). 

Methodological assessment is summarized in Table 6.

## 4. Discussion

This systematic review aimed to synthetize the evidence on ActiGraph GT9X step-counting and energy-expenditure measurement criterion validity. A total of eight articles were included in this systematic review. Among them, the accuracy of ActiGraph GT9X devices for step counting was investigated in seven studies and for energy expenditure measurement in one study. Due to the scarcity of available studies, but also to the heterogeneity of experimental protocols used (i.e., settings, duration of acquisition, device sample rate, data processing, validity indices, and population), quantitative evaluation was not possible and only a qualitative synthesis was carried out. 

We found that: (1) The ActiGraph GT9X generally underestimates steps when compared with the criterion; (2) the criterion validity of ActiGraph GT9X in measuring steps seems to be influenced by gait speed, device placement, filtering process, and monitoring conditions; and (3) there is a lack of evidence regarding the accuracy of step counting in free-living conditions and regarding energy-expenditure estimation using the ActiGraph GT9X device (Table 2).

We further found that, based on the qualitative synthesis of included studies, the GT9X globally underestimated steps when compared with the criterion measure. 

Comparing our results with the current literature on other devices was challenging, because reviews [24,25] do not always distinguish between different types of validity (criterion, construct, and content). Chevance and colleagues [23] investigated the criterion validity of wrist-wearable Fitbit devices. These authors found results that were consistent with our findings, with 40% of studies included in their review reporting underestimation by the Fitbits compared with criterion measures for steps.

On other hand, our findings are consistent with the results from Ngueleu and collaborators [16] for other Actigraph devices versus criterion validity. In that systematic review, the authors calculated the MPE of ActiGraph GT3X or wGT3X (n = 5) devices and found that across 24 comparisons, 92% underestimated steps (MPE ranged from −58% to 0.9%) counted by their criterion measure regardless of the wearing position, walking speed, and population. Of these, 13 (54%) were within ±3% measurement error and 11 (46%) were below −3% measurement error.

We also found that walking speed influences the accuracy of ActiGraph GT9X devices during both treadmill [36,38,39] and overground walking [33]. No studies conducted on treadmill walking reported an acceptable accuracy (MAPE ≤ 3%) at a speed below 0.88 m/s. 

Consistent with our results, Ngueuleu and colleagues [16] reported the effect of walking speed on ActiGraph device accuracy and concluded that none of the included studies demonstrated an acceptable estimation of steps below 0.9 m/s.

Considering overground walking, Anens and collaborators [33] found acceptable accuracy for individuals with multiple sclerosis at slow (0.73 m/s), comfortable (1.02 m/s), and fast (1.30 m/s) self-selected speeds with an LFE filter, and at fast self-selected speed with NF. 

A possible explanation could involve how steps are detected by the ActiLife software. Indeed, steps are counted when acceleration recorded by the ActiGraph GT9X exceeds the acceleration amplitude threshold [36]. This could explain why an underestimation of number of steps is observed at walking speeds, since low walking speeds produce lower accelerations that may be less likely to exceed the threshold for step detection. Moreover, noise-to-signal ratio is increased at slow speeds and this could contribute to reduced device accuracy. 

We also found that there is an effect of wearing position on the accuracy of ActiGraph GT9X devices in treadmill walking [36,38,39] and in overground walking [37,39]. 

During treadmill walking, ankle-mounted devices led to a better estimate of number of steps compared with hip-mounted [39]. Similarly, hip placement resulted in a better step-count estimate than wrist placement [36,38]. This result is also consistent with the findings of Ngueuleu and collaborators on other ActiGraph devices [16]. This could be explained by the distance of the device from the center of mass and is consistent with previous works by our group comparing the accuracy of another ActiGraph device (GT3X) [42,43]. Indeed, the acceleration detected by devices placed closer to the body’s center of mass could better reflect the displacement of the whole body [42,43]. In addition, devices worn on the hip are less affected by accelerations caused by non-locomotor movements such as daily activities (e.g., cooking, brushing teeth) or during a walking task that involves the hands (e.g., walking with a phone) [44]. Regarding ankle-mounted devices, an ActiGraph GT9X placed at the ankle seems to display the most accurate step-count estimate at comfortable speed. This could again be explained by the increased detection capability of step-related accelerations, since the device is placed on the body part (i.e., the leg) that is primarily displaced during walking and should better capture gait events such as the ground impact of the foot [45]. 

Regarding the filtering process, Anens and collaborators [33] compared the effects of different filtering methods on the accuracy of the ActiGraph GT9X in multiple sclerosis patients during overground walking. These authors found that LFE was the most accurate filter at slow (0.73 m/s), comfortable (1.02 m/s), and fast (1.30 m/s) speeds, followed by MAVM and NF. Similarly, Ngueleu and colleagues [16] reported that accuracy in step counting was impacted by the filtering process applied in other ActiGraph devices. These authors emphasized that the LFE effect does not appear to be relevant for high-intensity movements [46], such as high walking speed during overground walking. Nevertheless, LFE seems to be useful to improve device accuracy in populations that have slow gait patterns, such as individuals with Parkinson disease [47], multiple sclerosis [33], stroke [48], obesity [49,50,51], or older adults [52].

In relation to experimental setting, it is of note that only one study [19] examined the accuracy of the ActiGraph GT9X for step counting in free-living conditions. This is consistent with the low numbers of published articles that were identified in the systematic review by Ngueleu and colleagues [16], including only two studies that investigated the accuracy of step counting on ActiGraph devices in free-living conditions using a StepWatch device (Modus Health, Inc., Washington, DC) as a criterion measure [53,54]. 

In the study by Toth and collaborators [19], the authors reported that LFE overestimated steps in real-world settings for heathy adults, particularly when devices were worn on the wrist [19]. Consistently, a previous study on the ActiGraph GT3X found that an LFE filter estimated more steps during a free-living day compared with NF [55]. This result could be explained by the fact that, in daily life activities (cooking, driving a car, etc.), arm movements are often unrelated to walking and, since LFE is a more sensitive filtering method than NF, the former could have been impacted more severely than the latter. To this end, studies in controlled settings such as treadmill or overground walking are useful to understand how gait parameters can affect step or energy-expenditure measurement accuracy, but these results cannot be directly extended to the actual conditions of use. However, on the other hand, evaluation of devices in free-living conditions is challenging, primarily due to the difficulty to produce acceptable criterion measures. For ActiGraph GT9X, researchers proposed recording every step across a day using video recording [19]. However, this method is time-consuming and impractical. Indeed, based on a recent review [56], at least 20 to 28 h of video examination and a minimum of two raters are required to produce an acceptable criterion measure for daily steps per participant. In France, based on local labor laws (35 h per week for 47 weeks), this duration represents at least 17 to 24 months of full work for a sample size of 30 participants. These findings suggested that an alternative criterion measure should be found to encourage evaluation of wearable devices in free-living settings and to explore inter-day variability more easily. To this end, Toth and collaborators [19] found that the StepWatch 3 device produced accuracy within 3% for 1-day recordings and could be considered as a valid alternative criterion measure for daily step counting. However, these results do not provide any information on the potential variation in device accuracy between activities. In the same way, if the accuracy of the GT9X is not the same according to the nature of the activity performed [39], we can hypothesize that the number of steps recorded by this device per day may be the result of steps really taken, false-positive steps, and false-negative steps. In accordance with a previous study [57], we suggest assessing the accuracy of this device in various activities of daily living (i.e., locomotion and non-locomotion activities) in order to better understand the source of device error. This could be achieved by re-analyzing existing data. As for energy expenditure, in our review, we found a lack of studies investigating the criterion validity of the ActiGraph GT9X, with only one study [35] focusing on this metric. The findings of this report suggest that the GT9X generally underestimated energy expenditure compared with indirect calorimetry. However, device placement and gait speed were demonstrated to have an effect on device accuracy. In fact, authors reported that underestimation of energy expenditure was generally consistent across different walking speeds and device placements, except when the GT9X was worn at the hip and participants walked at 4.8 km/h. Under these conditions, the device provided the closest estimate of energy expenditure compared with the criterion. This finding is consistent with a previous study on the ActiGraph GT3X [42] suggesting that hip placement was superior to wrist for energy-expenditure estimation, and this is in line with a previous systematic review [15]. However, another study [58] found that an ActiGraph GT3X worn at the hip overestimated energy expenditure while walking and underestimated it during jogging or running on synthetic soccer grass. Similar to step accuracy, the same hypotheses (i.e., easier detection of body displacement when the device is closer to the center of mass and reduced detection of non-locomotor-related movements) can be formulated to explain the difference in energy-expenditure measurement [42,43,44].

We recommend that future studies or programs that will use the ActiGraph GT9X to estimate steps or energy expenditure—either for measurement purposes or as an intervention tool to stimulate physical activity—should take into consideration the different factors that have been reported to affect its measurement properties in either positive or negative ways, namely, the targeted end-user population, the wearable activity tracker’s placement on the body, and the monitoring conditions (laboratory versus field settings), as well as the features of the wearable activity tracker’s hardware and software.

### Limitations and Perspectives

We acknowledge that our study has limitations. First, only studies written in English were included in this systematic review, which is a potential bias. The GT9X is a recent device (released in 2014) from ActiGraph. This may explain our second limitation, namely that only a low number of studies were included. The high level of heterogeneity prevented us drawing conclusive evidence, especially on energy expenditure. This should stimulate future studies assessing the accuracy of ActiGraph GT9X in estimating step count and energy expenditure, taking into consideration device placement, monitoring conditions, gait speed, and filtering methods. Moreover, more studies in free-living conditions are warranted to collect data on the validity and accuracy of the ActiGraph GT9X in the settings where the device are most likely to be used. Finally, we did not include information on the acceptability of devices. This is a critical aspect for implementing a new technology into clinical trials and everyday practice and future studies are needed to address this relevant issue.

## 5. Conclusions

In conclusion, the ActiGraph GT9X generally underestimated steps, particularly in controlled settings, whereas an overestimation of step count was observed more frequently in semi-free and free-living conditions. The validity and accuracy of GT9X for step count seem to be influenced by device placement, with ankle- and hip-mounted devices showing better accuracies; gait speed, with reduced accuracy at lower speed; and the filtering process, with highly sensitive filtering methods overestimating steps, particularly when the device is worn on the wrist and in uncontrolled conditions. Only a very limited number of studies investigated the criterion validity and accuracy of the ActiGraph GT9X for step count under free-living conditions and for energy expenditure, with the GT9X showing a general underestimation of the latter parameter. Given the limited number of included studies and their heterogeneity, the present review emphasizes the need for further validity studies of the ActiGraph GT9X Link across age groups, in different populations, and in both controlled and free-living settings in order to achieve a larger body of evidence that could guide the implementation of these devices into clinical practice and in clinical trials.

## Figures and Tables

**Table 1:** General information about the included studies, including authors, year of publication, country, title, journal, funding sources.

Author, Year	Country	Title	Journal	Main Objective	Funding
Toth et al., 2018 [19]	USA	Video-recorded validation of wearable step counters under free-living conditions	Medicine & Science in Sports & Exercise	To investigate the step-count accuracy of several consumer- and research-grade activity monitors across all waking hours of 1 day.	This study was not funded
John et al., 2018 [36]	USA	“What is a Step?” Differences in how a step is detected among three popular activity monitors that have impacted physical activity research	Sensors	To compare manually counted steps during treadmill walking with those from the hip-worn Digiwalker SW200 and Omron HJ720ITC, and steps from hip- and wrist-worn GT3X+ and GT9X monitors processed using ActiLife software.	Not mentioned
Ata et al., 2018 [34]	USA	Clinical validation of smartphone-based activity tracking in peripheral artery disease patients	Digital Medicine	To assess the feasibility of the 6MWT app, “VascTrac,” to serve as a platform for performing 6 min walking tests in patients with PAD by (1) evaluating the accuracy of the iPhone’s step- and distance-tracking algorithms in the peripherical artery disease population, and (2) assessing the concordance of the iPhone algorithms with the ActiGraph GT9X.	Spectrum Stanford Predictives and Diagnostics Accelerator and the Stanford Precision Health and Integrated Diagnostics Center
Tedesco et al., 2019 [39]	Ireland	Accuracy of consumer-level and research-grade activity trackers in ambulatory settings in older adults	Plos One	To investigate the validity of different activity trackers in the estimation of step count, distance walked, and heart rate across a number of walking/household/sedentary activities recreated in a lab environment in a cohort of older adults.	This publication developed from research supported by EU H2020 funded project ProACT under grant agreement No. 689996. Aspects of this work were supported in part by a research grant from Science Foundation Ireland (SFI) and co-funded under the European Regional Development Fund under Grant Number 13/RC/2077. Aspects of this work were supported in part by INTERREG NPA funded project SenDOC.
Ho et al., 2019 [35]	China	Correction of estimation bias of predictive equations of energy expenditure based on wrist/waist-mounted accelerometers	PeerJ	To modify the traditional EE estimation equation: Freedson VM3 Combination, 2011 (ActiGraph, 2018), which is suitable for devices worn on different parts of the body.	Ministry of Science and Technology, Taiwan, under Grant MOST 107-2410-H-179-007.
Lynn et al., 2020 [37]	USA	Step-counting validity of wrist-worn activity monitors during activities with fixed upper extremities	Journal for the Measurement of Physical Behaviour	To examine the step-counting accuracy of wrist-worn activity monitors (Fitbit Charge HR 2, ActiGraph, Apple Watch Series 4) during various functional physical activities that require walking with the upper extremities fixed.	Not mentioned.
Mora-Gonzalez et al., 2022 [38]	USA	A catalog of validity indices for step counting wearable technologies during treadmill walking: the CADENCE-adults study	International Journal of Behavioral Nutrition and Physical Activity	To expand a previously published child/youth catalog of validity indices to include adults (21–40, 41–60, and 61–85 years of age) assessed across a range of treadmill speeds (slow [0.8–3.2 km/h], normal [4.0–6.4 km/h], fast [7.2–8.0 km/h]), and device locations (ankle, thigh, waist, and wrist)	The CADENCE-adults study was supported by NIH NIA Grant 5R01AG049024.
Anens et al., 2023 [33]	Sweden	Validity and reliability of physical activity measures in multiple sclerosis	Physiotherapy Theory and Practice	To evaluate the validity and test–retest reliability of different measures of physical activity in patients with multiple sclerosis.	Norrbacka Eugeniastiftelsen; ALF funding; P. O. Zetterling Foundation.

**Table 2:** Schematic representation of population and experimental procedure of included studies. Colors are used to indicate mentioned information in each study (orange: population; light grey: sex; dark gray: BMI class; red: outcomes; green; settings; yellow: device positioning; blue: filtering methods). Within the same category, blank cells mean that the information was not mentioned. BMI: body mass index; LFE: low-frequency extension; MAVM: moving average vector magnitude; NF: normal filter.

Ref	Population					Gender		Body Mass Index				Outcomes		Settings				Wearing Position			Filter or Algorithm		
	Young adults [21–40 year]	Middle-aged adults [41–60 year]	Older adults [61–85 year]	Multiple sclerosis	Peripherical artery disease	Male	Female	Underweight <18.5	Normal [18.5–24.9]	Overweight [25–29.9]	Obese >30	Steps	Energy expenditure	Controlled over ground walking	Controlled treadmill walking	Semi free-living	Free-living	Hip	Wrist	Ankle	LFE	Normal filter	MAVM
Toth et al., 2018 [19]																							
John et al., 2018 [36]																							
Ata et al., 2018 [34]																							
Tedesco et al., 2019 [39]																							
Ho et al., 2019 [35]																							
Lynn et al., 2020 [37]																							
Mora-Gonzalez et al., 2022 [38]																							
Anens et al., 2023 [33]																							

**Table 3:** Basic demographic and anthropometric characteristics of the group participants in included studies. F: female; M: male; MAA: middle-aged adults [41–60 years]; NM: not mentioned; OA: older adults [61–85 years]; YA: young adults [21–40 years]. *: calculated from height and weight using the formula: weight/(height)^2^.

Author, Year	N (% Female)	Health Status	Age, Years	Weight, kg	Height, cm	BMI, kg/m^2^
Toth et al., 2018 [19]	12 (50%)	Healthy	35 ± 13	72.1 ± 20.5	170.7 ± 10.7	24.3 ± 4.4
John et al., 2018 [36]	20 (40%)	Healthy	26.7 ± 4.9	NM	NM	26.1 ± 3.5
Ata et al., 2018 [34]	114 (23%)	Peripherical artery disease	69.5 ± 13.1	79.6 (16.3)	172 ± 10	26.9 ± 4.7
Tedesco et al., 2019 [39]	18 (61%)	Healthy	F: 69.7 ± 2.4 M: 69 ± 3.2	F: 66.1 ± 4.25 M: 79.7 ± 4.46	F: 162.9 ± 5.8 M: 175.8 ± 4.8	F: 24.9 * M: 25.8 *
Ho et al., 2019 [35]	90 (46%)	Healthy	22.90 ± 4.15	63.90 ± 12.06	168.05 ± 7.62	22.52 ± 3.25
Lynn et al., 2020 [37]	16 (50%)	Healthy	21.4 ± 1.1	70.0 ± 18.1	174 ± 10	22.8 ± 3.7
Mora-Gonzalez et al., 2022 [38]	YA: 80 (50%) MAA: 80 (50%) OA: 98 (49%)	Healthy	30.1 ± 5.8 50.2 ± 5.9 72.6 ± 6.9	72.5 ± 14.0 76.3 ± 14.2 72.7 ± 12.6	170.7 ± 9.2 171.0 ± 9.2 167.3 ± 8.5	24.8 ± 3.4 26.0 ± 4.0 25.9 ± 3.5
Anens et al., 2023 [33]	30 (70%)	Multiple sclerosis	49.2 ± 14.0	NM	NM	26.6 ± 5.5
Average	-	-	49.4 ± 20.1	73.1 ± 5.3	169.9 ± 2.3	25.3 ± 1.5

**Table 4:** ActiGraph GT9X settings used in included studies. IMU: inertial measurement unit; LFE: low-frequency extension; MAVM: moving average vector magnitude; NF: normal filter.

Authors, Year	Sampling Frequency	Extraction Methods	Epochs	Actilife Version
Toth et al., 2018 [19]	Not mentioned	LFE NF MAVM	60 s epochs	v6.13.1
John et al., 2018 [36]	80 Hz	Not mentioned	Not mentioned	v6.13.3
Ata et al., 2018 [34]	100 Hz (IMU)	LFE	1 s epochs	v6.13.3
Tedesco et al., 2019 [39]	Not mentioned	NF	Not mentioned	Not mentioned
Ho et al., 2019 [35]	30 Hz	Not mentioned	10 s epochs	v6.12.1
Lynn et al., 2020 [37]	Not mentioned	MAVM	Not mentioned	v6
Mora-Gonzalez et al., 2022 [38]	80 Hz	NF	1 s epochs	v6.11.8
Anens et al., 2023 [33]	90 Hz	LFE NF MAVM	1 s epochs	v6

**Table 5:** Summary of included studies examining the validity of step and energy-expenditure measurement using the ActiGraph GT9X. All speeds were converted in m s^−1^ to two decimal places. NM: not mentioned; IMU: inertial measurement unit; F: female; M: male; MAA: middle-aged adults [41–60 years]; OA: older adults [61–85 years]; SF: sampling frequency; YA: young adults [21–40 years].

Author, Year	N (% Female)	Participants’ Age, Year	Positioning of ActiGraph GT9X	ActiGraph Orientation (Attachment Bracket)	Signal Processing	Criterion Measure	Walking Task	Duration of Assessment
Toth et al., 2018 [19]	12 (50%)	35 ± 13	1 hip (right) 1 wrist (right) 1 wrist (left)	Hip: at the waistband, in line with the right anterior axillary line. Wrist: the device in the first position (closest to the hand): proximal to the ulnar styloid process. The device in the second position: proximal to the first device, but not touching it	NM	Video recording (≤2 observers)	NA	1 day
John et al., 2018 [36]	20 (40%)	26.7 ± 4.9	1 hip (right/left) 1 wrist (right/left)	Hip: in line with the anterior axillary line on the left and right hip Wrist: the most distal location on the left and right wrist	SF: 80 Hz	Direct observation	Laboratory: Treadmill: 11 speeds: 0.89 to 1.79 with 0.09 m/s increment	1 min
Ata et al., 2018 [34]	114 (23%)	69.5 ± 13.1	1 hip (right)	Hip: at the waistband	SF: 100 Hz IMU	Video recording	Overground: 6MWT: 100-feet	6 min
Tedesco et al., 2019 [39]	18 (61%)	F: 69.7 ± 2.4 M: 69 ± 3.2	1 ankle (dominant) 1 hip (dominant)	Hip: on the waist, midaxillary line	NM	Direct observation and/or video recording	Laboratory: Treadmill: 3 speeds: 0.28; 0.42 and 0.56 m/s Overground: Walking carrying a box Walking with rollator Walking upstairs Walking downstairs Semi free living: Dusting	3 min
Ho et al., 2019 [35]	90 (46%)	22.90 ± 4.15	1 hip (right) 1 wrist (non-dominant)	Hip: on the midaxillary line (soft elastic belt)	SF: 30 Hz	Indirect calorimetry, Vmax Encore 29 system; VIASYS Healthcare Inc, Yorba Linda, CA, USA	Laboratory: Treadmill: 5 speeds: 0.28; 1.78; 2.22; 2.69; and 3.14 m/s	>3 min
Lynn et al., 2020 [37]	12 (50%)	21.4 ± 1.1	1 wrist (right) 1 wrist (left)	Most proximally on both wrists	NM	Video recording (≤2 observers)	Laboratory: Treadmill: 1 speed: 1.33 m/s Overground: Walking with a baby doll on the left and right hip at 1.34 m/s Walking while holding grocery bags in each hand at 1.34 m/s Walking with hands fixed on a stroller at 1.34 m/s Running with hands fixed on a stroller at 2.78 m/s	1 min
Mora-Gonzalez et al., 2022 [38]	Total: 258 (50%) YA: 80 (50%) MAA: 80 (50%) OA: 98 (49%)	Total: 52.5 ± 18.7 YA: 30.1 ± 5.8 MAA: 50.2 ± 5.9 OA: 72.6 ± 6.9	1 hip (right) 1 wrist (non-dominant)	NM	SF: 80 Hz	Direct observation and/or video recording	Laboratory: Treadmill: 10 speeds: 0.28 to2.68 with 0.278 m/s increment	5 min
Anens et al., 2023 [33]	30 (70%)	49.2 ± 14.0	1 hip (right)	At the mid-thigh line	SF: 90 Hz	Video recording (≤2 observers)	Overground: 8 m cones 3 speeds: Slow: 0.73 m/s Comfortable: 1.02 m/s Fast: 1.30 m/s	5 min

**Table 6:** Quality of included studies with scores across reporting, external validity, and internal validity sub-scales. SD: Standard deviation.

Author, Year	Reporting (/9)	External Validity (/3)	Internal Validity (/7)	Total Score (/19)	Relative Score (%)
Toth et al., 2018 [19]	7	1	5	13	68%
John et al., 2018 [36]	6	1	5	12	63%
Ata et al., 2018 [34]	7	1	5	13	68%
Tedesco et al., 2019 [39]	6	1	5	12	63%
Ho et al., 2019 [35]	6	1	5	12	63%
Lynn et al., 2020 [37]	6	1	5	12	63%
Mora-Gonzalez et al., 2022 [38]	8	1	5	14	74%
Anens et al., 2023 [33]	9	1	5	15	79%
Mean ± SD	6.9 ± 1.1	1.0 ± 0.0	5.0 ± 0.0	12.9 ± 1.1	67.6 ± 6.0%

## Data Availability

Data sharing is not applicable to this article.

## Supplementary Materials

The following supporting information can be downloaded at: https://www.mdpi.com/article/10.3390/s24030825/s1, Table S1: Modified Evaluation Template for Assessing Quality of Physical Activity Validation Studies; Table S2: Main findings of studies examining ActiGraph GT9X step counting validity; Table S3: Main findings of studies examining ActiGraph GT9X energy expenditure validity; Table S4: Individual quality items of included studies.
