# Supplementary material for: Current Knowledge about ActiGraph GT9X Link Activity Monitor Accuracy and Validity in Measuring Steps and Energy Expenditure: A Systematic Review
Source: Sensors (Basel). 2024 Jan 26;24(3):825. doi: 10.3390/s24030825 (PMC10857518; doi:10.3390/s24030825)
Supplement: Supplementary file 1 [file sensors-24-00825-s001.zip › Supplementary Table S1.pdf]

Supplementary Table S1. Modified Evaluation Template for Assessing Quality of Physical Activity Validation Studies: The Hagströmer Bowles Physical Activity/Sedentary Behavior Questionnaire Checklist used in Wu et al. (2022) [26] and O'Brien et al. (2022) [27].

**Subscale A: Reporting**
1. Is the hypothesis/aim/objective of the study clearly described?
2. Are the operational definitions of main physical activity constructs to be validated clearly described in the Introduction or Methods section? If the main constructs (for example, frequency, duration, intensity, volume, type, domain for physical activity or type of sedentary behavior etc.) are first mentioned in the Results section, the question should be answered no.
3. Are the characteristics of the participants included in the study clearly described? Inclusion and/or exclusion criteria should be listed.
4. Are the distributions of principal confounders clearly described? A list of principal confounders is provided. At a minimum, distributions of sex, age, and education should be presented. Other confounders, such as BMI or race/ ethnicity, may be important depending on the population.
5. Are the methods of administration for the activity measure and the reference measure clearly described?
6. Are the methods of data reduction for the activity measure and the reference measure clearly described?
7. Have the characteristics of participants with missing, incomplete, and/or invalid data been described? This should be answered yes where there were no participant exclusions based on missing or poor data or where the number of exclusions were so small that findings would be unaffected by inclusion. This should be answered no, where a study does not describe or report the number of participants excluded based on missing or poor data.
8. Does the study provide information about the variability in the data for the main physical activity constructs? Simple descriptive statistics (eg, means, standard deviations, medians, value ranges, frequencies) should be reported for both the activity measure and the reference measure so the reader can check the major analyses and conclusions. This question does not cover statistical tests which are considered below.

9. Have limits of agreement and/or confidence intervals been reported for the main analyses?
**Subscale B: External Validity**
10. Were the individuals asked to participate in the study representative of the entire population from which they were recruited? The study must identify the source population for the sample and describe how the sample was selected. Samples would be representative if they comprised the entire source population, an unselected group of consecutive patients in a clinical setting, or a random sample. Random sampling is only feasible where a list of all members of the relevant population exists. Where a study does not report the proportion of the source population from which the samples are derived, the question should be answered as unable to determine.
11. Were those participants who were enrolled in the study representative of the entire population from which they were recruited? The proportion of those excluded after recruitment should be stated. Evidence that the enrolled participants are representative would include demonstrating that the distribution of the principal confounding factors was the same between the study participants and recruited individuals who were not enrolled (excluded and/or nonresponders).
12. Was the mode of administration representative of the procedures applied for similar study designs? Was it tested in free-living conditions?
**Subscale C: Internal Validity**
13. Was an attempt made to blind research staff to the activity levels or characteristics of the participants to avoid biasing the results? For studies where the research staff had no way of knowing the activity levels or characteristics of the participants, or where the self-report measure was administered by self-completion without aid, prompting, or verification by research staff this should be answered yes.
14. Does the reference measure assess the physical activity construct(s) of interest with greater accuracy than objective activity monitors? For studies where the validity of the reference measure was clearly described, the question should be answered yes. For studies which refer to other work that demonstrates the reference measure is more valid compared with activity monitors then question should be answered yes. For studies that used a reference measure that is not a direct

measure of physical activity (e.g., body composition, respiratory function, cholesterol) or concurrent validity (another monitor) the question should be answered no.
15. Did the monitor and the reference measure assess physical activity in the same time frame? If the monitor measure assessed physical activity engaged in over a long period of time during the past, such as past month or past year, the reference measure should have been administered repeatedly over this interval to account for variation in activity.
16. Was compliance with the measurement protocol acceptable? Where there was a high level of noncompliance (>20% of sample) or difference in the level of compliance by principal confounder subgroups the question should be answered no. For studies where the effect of any misclassification was likely to bias measures of agreement to the null, the question should be answered yes.
17. Were the statistical tests used appropriate to assess agreement for the main physical activity constructs between the monitor measure and the reference measure? The statistical techniques used must be appropriate to the data. If the self-report and reference measures assess physical activity constructs in the same units (e.g., minutes per week) then the analysis should provide an indication of over- or underestimation by self-report (e.g., Bland-Altman method). If the measures assess different constructs (e.g., minutes per week versus energy expenditure) then correlation coefficients should be provided, or responses to the self-report should be divided into quantiles and reference measure means should be compared. For categorical data sensitivity and specificity should be calculated.
18. Were all analyses planned at the outset of the study? If data dredging was used, it was identified and made clear. Any analyses that had not been planned at the outset of the study should be clearly indicated. If no retrospective unplanned subgroup analyses were reported, then answer yes.
19. Did the study appear to have sufficient sample size to assess agreement? If sample sizes were calculated to detect a level of correlation or agreement for principal confounder subgroups (e.g., men and women separately) then the question should be answered as yes.
**Removed Questions from Original Questionnaire**

Originally Q5: For studies validating an existing self-report measure has the original source of the measure been cited? For studies validating a modified version an existing self-report measure, has the original source of the measure been cited, and have the modifications been clearly described? If the study is validating a new measure the question should be answered yes. Modifications include cultural adaptation, language translation, change in question order or wording, and change in scoring protocol from the original source. If the study is validating an existing measure developed for a population culturally or linguistically different from the study population without reporting whether the measure was adapted/translated the question should be answered as unable to determine.
Originally Q14: Was an attempt made to minimize altered physical activity behavior by the participant in the use of the validity measures? For studies where self-report physical activity was assessed by recall and/or participant burden for collecting the reference measure was low this should be answered yes.
Originally Q19: Was reproducibility of the main physical activity construct(s) reported for the self-report measure? For test-retest reliability was the time interval between self-report measure administrations appropriate?

Note: All questions were scored as: Yes = 1, No = 0, Cannot Determine = 0.

This quality checklist was adapted from the The Hagströmer Bowles Physical Activity/Sedentary Behavior Questionnaire Checklist to apply to an objective monitor. Original questions that did not apply to this study (n=3) of the original 22 question checklist were removed.

Citation: Maria Hagströmer, Barbara E. Ainsworth, Lydia Kwak, and Heather R. Bowles. 2012. A Checklist for Evaluating the Methodological Quality of Validation Studies on Self-Report Instruments for Physical Activity and Sedentary Behavior. Journal of Physical Activity and Health. 9(1), S29-S3
