# Supplementary material for: Current Knowledge about ActiGraph GT9X Link Activity Monitor Accuracy and Validity in Measuring Steps and Energy Expenditure: A Systematic Review
Source: Sensors (Basel). 2024 Jan 26;24(3):825. doi: 10.3390/s24030825 (PMC10857518; doi:10.3390/s24030825)
Supplement: Supplementary file 1 [file sensors-24-00825-s001.zip › Supplementary Table S2.pdf]

Supplementary Table S2. Main findings of studies examining ActiGraph GT9X step counting validity.

Author, year	Criterion measure	Criterion validity indices	Wearing position	Outcomes
**Toth et al., 2018 [19]**	Video recording (≤2 observers)<br><br>« Researchers were trained in step counting by the principal investigator, and their step counting accuracy was assessed using practice videos. Training included learning the definition of a step, discussing different types of steps (e.g., forward, sideways, backwards, turning), and instructions on how to use the hand tally counter. A step was defined as picking the foot up off the ground and placing it down in a new location (19). Steps taken with both the left and right feet were counted using a dual-unit hand tally device and were tabulated	MAPE, MD, 95% CI, % of step counted, Pearson's r, t-test	Hip (right)<br>Wrist (dominant)<br>Wrist (non-dominant)	<u>MAPE, %</u><br>Hip (right):<br>**LFE: 28.1, p<0.05**<br>**NF: 30.8, p<0.05**<br>**MAVM: 30.1, p<0.05**<br><br>Wrist (dominant):<br>**LFE: 119.7, p<0.05**<br>NF: 30.4, p>0.05<br>MAVM: 25.1, p>0.05<br><br>Wrist (non-dominant):<br>**LFE: 95.7, p<0.05**<br>NF: 25.0, p>0.05

separately. Before reviewing the GoPro video recordings of participants, researchers were required to count steps for two training videos and report values within 5% of the primary investigator's counts. Before reviewing any participant videos, researchers signed a confidentially statement. The participant videos were downloaded from the microSD cards to a laptop computer. The videos were processed with a function that placed embedded date and time stamp on the lower left-hand corner of each video. They then were cut into 10-min segments for distribution to researchers. At the conclusion of the study, a total of 1079 10-min video segments were collected. This equated to approximately 178 h of video. Each 10-min video segment was independently counted by	MAVM: 28.6, p>0.05<br><br><u>MD, step</u><br>Hip (right):<br>**LFE: 2880.6, p<0.05**<br>**NF: -3491.2, p<0.05**<br>**MAVM: -3312.3, p<0.05**<br><br>Wrist (dominant):<br>**LFE: 11157.7, p<0.05**<br>NF: 1530.6, p>0.05<br>MAVM: -1323.4, p>0.05<br><br>Wrist (non-dominant):<br>**8910.8 (LFE:), p<0.05**<br>NF: 203.5, p>0.05<br>MAVM: -2148.5, p>0.05

two researchers, and step counts were then compared. If the step counts per 10-min segment differed by more than 5% (or six steps), the video was recounted by a third researcher. The two closest step counts were then averaged to determine the step count for the video segment. The total step count from all waking hours was found by summing the steps from all 10-min video segments per each participant. » (Toth et al., 2018, p. 3)	<u>Pearson's r</u><br>Hip (right):<br>LFE: 0.965<br>NF: 0.889<br>MAVM: 0.922<br><br>Wrist (dominant):<br>LFE: 0.780<br>NF: 0.792<br>MAVM: 0.802<br><br>Wrist (non-dominant):<br>LFE: 0.831<br>NF: 0.787<br>MAVM: 0.781

	<u>MPE, % (calculated with group values)</u><br><br>Hip (right):<br>LFE: 20,9<br>NF: -47,0<br>MAVM: 43.5<br><br>Wrist (dominant):<br>LFE: 50.5<br>NF: 12.3<br>MAVM: -13.8<br><br>Wrist (non-dominant):<br>LFE: 44.9<br>NF: 1.8<br>MAVM: -24.5

**John et al., 2018 [36]**	Direct observation<br><br>« A researcher manually counted steps (criterion) during the protocol. » (John et al., 2018, p. 4)	MD, 95% CI, significant test	Hip (right/left)<br>Wrist (right/left)	<u>MD (95% CI), step:</u><br>Hip (right/left):<br>**Treadmill, 0.89 m/s: 58 (50-67), p<0.05**<br>**Treadmill, 0.98 m/s: 63 (52-74), p<0.05**<br>**Treadmill, 1.07 m/s: 62 (53-72), p<0.05**<br>**Treadmill, 1.16 m/s: 64 (55-73), (p>0.05)**<br>**Treadmill, 1.25 m/s: 66 (55-76), p<0.05**<br>**Treadmill, 1.34 m/s: 64 (53-75), p<0.05**<br>**Treadmill, 1.43 m/s: 63 (51-75), p<0.05**<br>**Treadmill, 1.52 m/s: 60 (48-73),**

	**$p<0.05$**<br>**Treadmill, 1.61 m/s: 58 (45-70), $p<0.05$**<br>**Treadmill, 1.70 m/s: 56 (41-70), $p<0.05$**<br>**Treadmill, 1.79 m/s: 55 (42-68), $p<0.05$**<br><br>Wrist (right/left):<br>**Treadmill, 0.89 m/s: 68 (60-77), $p<0.05$**<br>**Treadmill, 0.98 m/s: 66 (57-74), $p<0.05$**<br>**Treadmill, 0.98 m/s: 66 (58-73), $p<0.05$**<br>**Treadmill, 1.16 m/s: 68 (58-73), $p<0.05$**

Treadmill, 1.25 m/s: 68 (60-76), $p<0.05$

Treadmill, 1.34 m/s: 67 (61-74), $p<0.05$

Treadmill, 1.43 m/s: 68 (62-74), $p<0.05$

Treadmill, 1.52 m/s: 69 (61-76), $p<0.05$

Treadmill, 1.61 m/s: 68 (62-74), $p<0.05$

Treadmill, 1.70 m/s: 71 (65-77), $p<0.05$

Treadmill, 1.79 m/s: 72 (65-78), $p<0.05$

MPE, % (calculated with group values)

Hip (right/left):

Treadmill, 0.89 m/s: -37,6

Treadmill, 0.98 m/s: -24,2

Treadmill, 1.07 m/s: -16,7

Treadmill, 1.16 m/s: -15,1

Treadmill, 1.25 m/s: -13,4

Treadmill, 1.34 m/s: -11,1

Treadmill, 1.43 m/s: -6,8

Treadmill, 1.52 m/s: -11,1

Treadmill, 1.61 m/s: -5,0

Treadmill, 1.70 m/s: -6,3

Treadmill, 1.79 m/s: -6,3

Wrist (right/left):

Treadmill, 0.89 m/s: -53,8

				**Treadmill, 0.98 m/s:** -55,8<br>**Treadmill, 1.07 m/s:** -55,9<br>**Treadmill, 1.16 m/s:** -56,6<br>**Treadmill, 1.25 m/s:** -57,1<br>**Treadmill, 1.34 m/s:** -54,7<br>**Treadmill, 1.43 m/s:** -52,1<br>**Treadmill, 1.52 m/s:** -49,2<br>**Treadmill, 1.61 m/s:** -43,0<br>**Treadmill, 1.70 m/s:** -40,5<br>**Treadmill, 1.79 m/s:** -38,6
**Ata et al., 2018 [34]**	Video-recording<br><br>« To establish our reference standard for steps, one of the trained clinical coordinators manually counted steps for each participant. To establish each coordinator's step counting error, we performed five videotaped trials in the same hallway under the same walk test	MPE, Bland-Altman plots	Hip (right)	Hip (right):<br>MPE, %:<br>−3.1 ± 10.3

conditions. During each trial, the coordinators manually counted number of steps taken by the test subject. The videos were then analyzed in slow motion by two different raters in order to determine the true number of steps taken in each trial. Using the videotaped trial steps as the ground truth, we were able to quantify each coordinator's accuracy. In order to participate in manual step counting during the study, coordinators were required to have a mean percent error of one percent or less. » (Ata et al., 2018, p. 7)

"A separate validation study to evaluate the accuracy of the eight manual step counters involved in the study was performed. Each step counter, i.e., rater, counted steps in five videotaped trials and their average for the five

	trials was calculated. The percent error for each rater was then tabulated using the number of steps measured in the video as ground truth. The mean steps measured in the video was 593 steps. » (Ata et al., 2018, Supplementary information)			
**Tedesco et al., 2019 [39]**	Direct observation and/or video-recording<br>« Steps were measured during all activities with direct visualization and/or video-recording as reference. » (Tedesco et al., 2019, p. 7)	MAPE, MPE, MAD, MAE, RMSE, ICC 2,1 (95% CI)	Hip (dominant)<br>Ankle (right)	<u>MAPE, %:</u><br>Hip (dominant):<br>Treadmill, 1 km/h: 87.05<br>Treadmill, 1.5 km/h: 77.20<br>Treadmill, 2 km/h: 62.99<br>Climb up the stairs: 11.87<br>Go down the stairs: 10.90<br>Carry a box: 16.39<br>Dusting: 89.81<br>Rollator: 84.38

	Ankle (right):<br><br>Treadmill, 1 km/h: 51.20<br><br>Treadmill, 1.5 km/h: 31.78<br><br>Treadmill, 2 km/h: 8.87<br><br>Climb up the stairs: 5.81<br><br>Go down the stairs: 43.30<br><br>Carry a box: 5.36<br><br>Dusting: 90.34<br><br>Rollator: 45.96<br><br><br>MPE, %:<br><br>Hip (dominant):<br><br>Treadmill, 1 km/h: -87.05 ± 18.58<br><br>Treadmill, 1.5 km/h: -76.37 ± 25.47<br><br>Treadmill, 2 km/h: -62.53 ± 29.19<br><br>Climb up the stairs: -7.81 ± 13.77<br><br>Go down the stairs: -9.53 ± 9.12

Carry a box: -12.67 ± 17.01

Dusting: -89.81 ± 14.23

Rollator: -84.38 ± 18.51

Ankle (right):

Treadmill, 1 km/h: -50.91 ± 33.18

Treadmill, 1.5 km/h: -31.02 ± 28.66

Treadmill, 2 km/h: -7.77 ± 12.61

Climb up the stairs: -3.01 ± 7.34

Go down the stairs: -30.37 ± 49.99

Carry a box: -1.81 ± 10.31

Dusting: -90.34 ± 17.2

Rollator: -45.96 ± 37.67

<u>MAD, step:</u>

Hip (dominant):

Treadmill, 1 km/h: 14.00

Treadmill, 1.5 km/h: 31.50

Treadmill, 2 km/h: 64.00

Climb up the stairs: 3.00

Go down the stairs: 3.00

Carry a box: 35.00

Dusting: 4.00

Rollator: 18.00

Ankle (right):

Treadmill, 1 km/h: 78.50

Treadmill, 1.5 km/h: 76.00

Treadmill, 2 km/h: 28.00

Climb up the stairs: 3.00

Go down the stairs: 22.00

Carry a box: 19.50

Dusting: 0.00

	Rollator: 90.00<br><br><u>MAE, step:</u><br>Hip (dominant):<br>Treadmill, 1 km/h: 207.91<br>Treadmill, 1.5 km/h: 186.94<br>Treadmill, 2 km/h: 161.50<br>Climb up the stairs: 6.39<br>Go down the stairs: 5.97<br>Carry a box: 48.81<br>Dusting: 80.64<br>Rollator: 170.67<br><br>Ankle (right):<br>Treadmill, 1 km/h: 109.03<br>Treadmill, 1.5 km/h: 71.26<br>Treadmill, 2 km/h: 22.22

Climb up the stairs: 3.00

Go down the stairs: 23.72

Carry a box: 16.00

Dusting: 80.58

Rollator: 83.30

RMSE:

Hip (dominant):

Treadmill, 1 km/h: 217.11

Treadmill, 1.5 km/h: 193.80

Treadmill, 2 km/h: 176.86

Climb up the stairs: 8.50

Go down the stairs: 6.68

Carry a box: 61.60

Dusting: 84.23

Rollator: 173.75

Ankle (right):

Treadmill, 1 km/h: 124.18

Treadmill, 1.5 km/h: 91.44

Treadmill, 2 km/h: 35.11

Climb up the stairs: 4.36

Go down the stairs: 31.75

Carry a box: 29.10

Dusting: 83.96

Rollator: 102.44

<u>ICC 2,1 (95% CI):</u>

Hip (dominant):

Treadmill, 1 km/h: 0.11 (-0.04 0.39)

Treadmill, 1.5 km/h: 0.17 (-0.05 0.50)

Treadmill, 2 km/h: 0.18 (-0.08 0.50)

Climb up the stairs: -0.12(-0.36 0.18)

Go down the stairs: 0.40 (<-0.01 0.67)

				Carry a box: 0.01 (-0.02 0.06)<br>Dusting: 0.01 (-0.02 0.06)<br>Rollator: 0.10 (-0.02 0.36)<br><br>Ankle (right):<br>Treadmill, 1 km/h: 0.51 (-0.10 0.82)<br>Treadmill, 1.5 km/h: 0.52(-0.05 0.80)<br>Treadmill, 2 km/h: 0.83 (0.56 0.93)<br>Climb up the stairs: 0.32 (-0.01 0.59)<br>Go down the stairs: 0.01 (-0.23 0.31)<br>Carry a box: 0.66 (0.42 0.81)<br>Dusting: 0.02 (-0.02 0.11)<br>Rollator: 0.41 (-0.11 0.73)
**Lynn et**	Video recording (≤2 observers)	MD, MPE	Wrist (right)	<u>MD, step:</u>

**al., 2020 [37]**	« Two researchers watched each video and used a hand tally counter to count and record the number of steps ambulated; agreement within 1 percent was necessary or steps were recounted. Of n =96 total videos (six trials per participant), 15 videos were recounted (15.6%). A step was defined as picking the foot up off the ground and placing it on the ground in a new location (Toth, Park, Springer, et al., 2018). » (Lynn et al., 2020, p. 3)	Wrist (left)	Wrist (right):<br>Treadmill walking holding handrails, 4.8 km/h: -50 ± 88, p<0.05<br>Stroller jogging, 10.5 km/h: -222 ± 159, p<0.05<br>Stroller walking, 4.8 km/h: -45 ± 87, p<0.05<br>Baby right walking, 4.8 km/h: -304 ± 46, p<0.05<br>Grocery walking, 4.8 km/h: -327 ± 31<br><br>Wrist (left):<br>Treadmill walking holding handrails, 4.8 km/h: -35 ± 68, p<0.05<br>Stroller jogging, 10.5 km/h: -78 ± 134, p<0.05<br>Stroller walking, 4.8 km/h: -19 ± 59,

$p<0.05$

Baby left walking, 4.8 km/h: -320 ± 20

Grocery walking, 4.8 km/h: -304 ± 57

MPE, %:

Wrist (right):

Treadmill walking holding handrails, 4.8 km/h: -84.7 ± 27.2

Stroller jogging, 10.5 km/h: -54.4 ± 32.4

Stroller walking, 4.8 km/h: -86.5 ± 26.3

Baby right walking, 4.8 km/h: -7.4 ± 13.5

Grocery walking, 4.8 km/h: -0.4 ± 8.6

Wrist (left):

				Treadmill walking holding handrails, 4.8 km/h: -89.1 ± 21.1<br>Stroller jogging, 10.5 km/h: -83.8 ± 28.2<br>Stroller walking, 4.8 km/h: -94.3 ± 17.9<br>Baby left walking, 4.8 km/h: -2.4 ± 5.9<br>Grocery walking, 4.8 km/h: -7.6 ± 15.8
**Mora-Gonzalez et al., 2022 [38]**	Direct observation and/or video-recording<br><br>« The criterion measure of steps taken was directly observed and hand-tally counted. The method for assessing treadmill stepping was rarely problematic, likely because this was the sole assignment of one research technician during the treadmill test but also because the	MAPE, MPE, CV	Hip (right)<br>Wrist (non-dominant)	**Young Adults (21-40 yr)**<br><u>MAPE, %:</u><br>Hip (right):<br>Treadmill, 0.8 km/h: 98,31 ± 3,96<br>Treadmill, 1.6 km/h: 80,93 ± 19,60<br>Treadmill, 2.4 km/h: 50,08 ± 24,87<br>Treadmill, 3.2 km/h: 9,14 ± 15,23

steps taken were largely rhythmic and predictable (except for the very few steps taken at the beginning and end of a bout) and the observed movements were reinforced with the audible sound made when the foot hit the treadmill band. We also aimed a video camera at the participant's feet during the test to provide a redundant copy of the event for verification purposes as needed. Our practice was that when the responsible research technician self-disclosed miscounting or the value reported was immediately identified as unusual or unexpected (i.e., higher or lower than expected given the preceding bout and/or recorded bout speed), the step count for that particular bout was verified and corrected as needed using the video file immediately	Treadmill, 4.0 km/h: 3,41 ± 8,34<br>Treadmill, 4.8 km/h: 4,24 ± 13,57<br>Treadmill, 5.6 km/h: 3,13 ± 8,22<br>Treadmill, 6.4 km/h: 3,73 ± 9,48<br>Treadmill, 7.2 km/h: 6,23 ± 12,31<br>Treadmill, 8.0 km/h: 19,22 ± 18,04<br><br>Wrist (non-dominant):<br>Treadmill, 0.8 km/h: 78,03 ± 17,25<br>Treadmill, 1.6 km/h: 67,14 ± 16,10<br>Treadmill, 2.4 km/h: 72,72 ± 14,45<br>Treadmill, 3.2 km/h: 65,72 ± 17,32<br>Treadmill, 4.0 km/h: 55,93 ± 13,87<br>Treadmill, 4.8 km/h: 52,57 ± 10,37<br>Treadmill, 5.6 km/h: 50,58 ± 8,45<br>Treadmill, 6.4 km/h: 49,94 ± 7,51<br>Treadmill, 7.2 km/h: 47,82 ± 9,66

following the testing session. During analysis, we had the opportunity to again examine rare cases of anomalous values (including questionable results compared to associated outputs from the multiple wearable technologies) by recounting steps on the video. If a discrepancy was found between the original logged value and the second viewing of the video, a third viewing was used to finalize the criterion value. We emphasize that this process was rarely required. » (Mora-Gonzalez et al., 2022, p. 3)		Treadmill, 8.0 km/h: 48,88 ± 0,68<br><br>MPE, %:<br>Hip (right):<br>Treadmill, 0.8 km/h: -98,31 ± 3,96<br>Treadmill, 1.6 km/h: -80,93 ± 19,60<br>Treadmill, 2.4 km/h: -50,06 ± 24,90<br>Treadmill, 3.2 km/h: -8,14 ± 15,79<br>Treadmill, 4.0 km/h: -1,26 ± 8,93<br>Treadmill, 4.8 km/h: -2,23 ± 14,05<br>Treadmill, 5.6 km/h: -0,86 ± 8,76<br>Treadmill, 6.4 km/h: -1,39 ± 10,10<br>Treadmill, 7.2 km/h: -3,92 ± 13,26<br>Treadmill, 8.0 km/h: -18,48 ± 19,05<br><br>Wrist (non-dominant):<br>Treadmill, 0.8 km/h: -78,03 ± 17,25

Treadmill, 1.6 km/h: -67,14 ± 16,10

Treadmill, 2.4 km/h: -72,72 ± 14,45

Treadmill, 3.2 km/h: -65,72 ± 17,32

Treadmill, 4.0 km/h: -55,93 ± 13,87

Treadmill, 4.8 km/h: -52,57 ± 10,37

Treadmill, 5.6 km/h: -50,58 ± 8,45

Treadmill, 6.4 km/h: -49,94 ± 7,51

Treadmill, 7.2 km/h: -47,61 ± 10,66

Treadmill, 8.0 km/h: -48,88 ± 0,68

Middle-Age Adults (41-60 yr)

MAPE, %:

Hip (right):

Treadmill, 0.8 km/h: 98,37 ± 4,31

Treadmill, 1.6 km/h: 77,78 ± 22,76

Treadmill, 2.4 km/h: 44,13 ± 25,56

Treadmill, 3.2 km/h: 9,55 ± 15,70

			Treadmill, 4.0 km/h: 3,72 ± 8,21<br>Treadmill, 4.8 km/h: 2,52 ± 3,72<br>Treadmill, 5.6 km/h: 2,39 ± 15,27<br>Treadmill, 6.4 km/h: 6,23 ± 3,27<br>Treadmill, 7.2 km/h: 3,88 ± 5,85<br>Treadmill, 8.0 km/h: 7,56 ± NA<br><br>Wrist (non-dominant):<br>Treadmill, 0.8 km/h: 86,57 ± 14,57<br>Treadmill, 1.6 km/h: 74,90 ± 19,83<br>Treadmill, 2.4 km/h: 75,69 ± 19,34<br>Treadmill, 3.2 km/h: 68,06 ± 19,37<br>Treadmill, 4.0 km/h: 56,42 ± 15,21<br>Treadmill, 4.8 km/h: 51,82 ± 12,90<br>Treadmill, 5.6 km/h: 48,35 ± 10,88<br>Treadmill, 6.4 km/h: 49,12 ± 9,36<br>Treadmill, 7.2 km/h: 50,52 ± 4,33

	Treadmill, 8.0 km/h: 49,49 ± NA<br><br>MPE, %:<br><br>Hip (right):<br><br>Treadmill, 0.8 km/h: -98,37 ±4,31<br><br>Treadmill, 1.6 km/h: -77,78 ±22,76<br><br>Treadmill, 2.4 km/h: -44,06 ±25,67<br><br>Treadmill, 3.2 km/h: -8,21 ±16,45<br><br>Treadmill, 4.0 km/h: -0,75 ±9,00<br><br>Treadmill, 4.8 km/h: 1,16 ±4,35<br><br>Treadmill, 5.6 km/h: 1,26 ±3,85<br><br>Treadmill, 6.4 km/h: -2,41 ±16,34<br><br>Treadmill, 7.2 km/h: -0,73 ±7,07<br><br>Treadmill, 8.0 km/h: -7,56 ± NA<br><br>Wrist (non-dominant):

Treadmill, 0.8 km/h: -86,57 ± 14,57

Treadmill, 1.6 km/h: -74,90 ± 19,83

Treadmill, 2.4 km/h: -75,69 ± 19,34

Treadmill, 3.2 km/h: -68,06 ± 19,37

Treadmill, 4.0 km/h: -56,42 ± 15,21

Treadmill, 4.8 km/h: -51,82 ± 12,90

Treadmill, 5.6 km/h: -48,35 ± 10,88

Treadmill, 6.4 km/h: -49,12 ± 9,36

Treadmill, 7.2 km/h: -50,52 ± 4,33

Treadmill, 8.0 km/h: -49,49 ± NA

Older Adults (61-85 yr)

MAPE, %:

Hip (right):

Treadmill, 0.8 km/h: 96,30 ± 12,28

Treadmill, 1.6 km/h: 86,05 ± 18,14

Treadmill, 2.4 km/h: 61,62 ± 21,60

Treadmill, 3.2 km/h: 22,80 ± 19,63

Treadmill, 4.0 km/h: 5,16 ± 6,92

Treadmill, 4.8 km/h: 3,74 ± 7,54

Treadmill, 5.6 km/h: 3,86 ± 6,40

Treadmill, 6.4 km/h: 4,29 ± 7,32

Treadmill, 7.2 km/h: 31,28 ± 26,48

Wrist (non-dominant):

Treadmill, 0.8 km/h: 82,53 ± 16,68

Treadmill, 1.6 km/h: 73,78 ± 18,49

Treadmill, 2.4 km/h: 69,11 ± 20,19

Treadmill, 3.2 km/h: 61,67 ± 20,31

Treadmill, 4.0 km/h: 54,42 ± 17,03

Treadmill, 4.8 km/h: 49,07 ± 15,58

Treadmill, 5.6 km/h: 46,56 ± 12,87

Treadmill, 6.4 km/h: 46,08 ± 11,16

Treadmill, 7.2 km/h: 45,90 ± 8,75

MPE, %:

Hip (right):

Treadmill, 0.8 km/h: -96,30 ± 12,28

Treadmill, 1.6 km/h: -86,01 ± 18,35

Treadmill, 2.4 km/h: -61,59 ± 21,67

Treadmill, 3.2 km/h: -22,52 ± 19,96

Treadmill, 4.0 km/h: -2,74 ± 8,20

Treadmill, 4.8 km/h: 0,03 ± 8,43

Treadmill, 5.6 km/h: 0,18 ± 7,49

Treadmill, 6.4 km/h: -0,69 ± 8,53

Treadmill, 7.2 km/h: -28,85 ± 30,34

Wrist (non-dominant):

Treadmill, 0.8 km/h: -82,53 ± 16,68

Treadmill, 1.6 km/h: -73,78 ± 18,49

Treadmill, 2.4 km/h: -69,11 ± 20,19

				Treadmill, 3.2 km/h: -61,67 ± 20,31<br>Treadmill, 4.0 km/h: -54,33 ± 17,32<br>Treadmill, 4.8 km/h: -49,00 ± 15,79<br>Treadmill, 5.6 km/h: -46,40 ± 13,45<br>Treadmill, 6.4 km/h: -46,08 ± 11,16<br>Treadmill, 7.2 km/h: -45,90 ± 8,75
**Anens et al., 2023 [33]**	Video recording (≤2 observers)<br><br>« The number of steps taken was counted by two registered physiotherapists through direct video observation and using hand-tally counters. A step was defined as a transition from the point of contact between one foot and the ground to the point of contact between the other foot and the ground. » (Anens et al., 2023, p. 6)	MeAPE, Wilcoxon test, Spearman's rho, Bland-Altman plots	Hip (right)	MeAPE, %:<br>Hip (right):<br>Comfortable, 1.02 m/s:<br>**Display: 10.9 (IQR 23.4, 0.2–100.0), p<0.001**<br>**NF: 22.0 (IQR 39.4, 0.4–99.2), p<0.001**<br>LFE: 0.9 (IQR 5.4, 0.0–39.5), p=0.425<br><br>Fast 1.30 m/s:

« The mean numbers of steps or seconds from the two observers were used for analysis. » (Anens et al., 2023, p. 6)<br><br>« The inter-rater reliability of the two raters of steps and time in different positions based on video analysis was calculated as ICC3,1 (two-way mixed model, absolute agreement, single measure). The correlations between the raters were very high with Intraclass Correlation Coefficient3,1 ICC3,1 1.000 (CI 1.000–1.000) for number of steps, […]» (Anens et al., 2023, p. 6)	**Display: 3.6 (IQR 16.4, 0.1–100.0), p=0.001**<br>**NF: 8.9 (IQR 28.4, 0.1–99.3), p<0.001**<br>LFE: 1.1 (IQR 3.4, 0.0–27.2), p=0.689<br><br>Slow, 0.73 m/s:<br>**Display: 39.5 (IQR 59.4, 2.5–100.0), p<0.001**<br>**NF: 47.3 (IQR 45.9, 4.4–99.1), p<0.001**<br>LFE: 2.6 (IQR 12.8, 0.1–49.4), p=0.411<br><br>All speed:

Display: 16.9 (IQR 20.6, 0.6–99.6), $p<0.001$

NF: 23.1 (IQR 31.1, 3.9–99.2), $p<0.001$

LFE: 1.9 (IQR 1.8, 0.1–35.2), $p=0.371$

< 1.1 m/s:

Display: 44.8 (IQR 57.0, 0.2–100.0), $p<0.001$

NF: 48.9 (IQR 46.2, 4.4–99.3), $p<0.001$

LFE: 3.1 (IQR 15.3, 0.1–48.7), $p=0.907$

⩾ 1.1 m/s:

Display: 5.7 (IQR 111.1, 0.1–37.9), p<0.001

NF: 10.1 (IQR 18.4, 0.1–49.4), p<0.001

LFE: 1.0 (IQR 3.3, 0.0–49.4), p=0.357

<u>Spearman's rho</u>

Hip (right):

Comfortable:

Display: 0.528, p=0.003

NF: 0.790, p<0.001

LFE: 0.925, p<0.001

Fast:

Display: 0.816, p<0.001

NF: 0.750, p<0.001

LFE: 0.924, p<0.001

Slow:

Display: 0.589, p<0.001

NF: 0.749, p<0.001

LFE: 0.851, p<0.001

All speeds:

Display: 0.566, p=0.001

NF: 0.741, p<0.001

LFE: 0.894, p=0.000

< 1.1 m/s:

Display: 0.707, p<0.001

NF: 0.796, p<0.001

LFE: 0.916, p<0.001

⩾ 1.1 m/s:

Display: 0.756, p<0.001

NF: 0.767, p<0.001

LFE: 0.906, p<0.001

MePE, % (calculated with group values)

Hip (right):

Comfortable, 1.02 m/s:

Display: -18.5

NF: -23.9

LFE: -6.4

Fast 1.30 m/s:

Display:-3.9

NF: -8.8

LFE: 0.0

Slow, 0.73 m/s:

		Display: -25.1<br>NF: -46.0<br>LFE: -4.0

Statistical significance was bolded. CI: confidence interval; CV: coefficient of variation; ICC: intraclass correlation; LFE: low frequency extension; MAE: mean absolute error; MAD: median absolute deviation; MAPE: mean absolute percentage of error; MAVM: moving average vector magnitude; MeAPE: median absolute percentage of error; MePE: median percentage of error; MD: mean difference; MPE: mean percentage of error; NF: normal filter; RMSE: root mean square error.
