# Supplementary material for: Current Knowledge about ActiGraph GT9X Link Activity Monitor Accuracy and Validity in Measuring Steps and Energy Expenditure: A Systematic Review
Source: Sensors (Basel). 2024 Jan 26;24(3):825. doi: 10.3390/s24030825 (PMC10857518; doi:10.3390/s24030825)
Supplement: Supplementary file 1 [file sensors-24-00825-s001.zip › Supplementary Table S3.pdf]

Supplementary Table S3. Main findings of studies examining ActiGraph GT9X energy expenditure validity.

Author, year	Criterion measure	Criterion validity indices	Wearing position	Outcomes
**Ho et al., 2019 [35]**	Indirect calorimetry<br><br>« Regarding the indirect calorimeter method, the Cardiopulmonary Exercise Testing System (Vmax Encore 29 System; VIASYS Healthcare Inc, Yorba Linda, CA) was adopted to conduct the metabolic criterion measure (CM).» (Ho et al., 2019, p. 3-4)	MAPE, Cohen's d, Pearson's r, ICC (NM)	Hip (right)<br>Wrist (left)	<u>MAPE, %:</u><br>Wrist (left):<br>**4.80 km/h: 21.43, $p<0.05$**<br>**6.42 km/h: 41.44, $p<0.05$**<br>**8.04 km/h: 52.03, $p<0.05$**<br>**9.66 km/h: 58.14, $p<0.05$**<br>**11.28 km/h: 63.86, $p<0.05$**<br><br>Hip (right):<br>4.80 km/h: 5.71 ($p>0.05$)<br>**6.42 km/h: 6.31, $p\leqslant0.05$**<br>**8.04 km/h: 4.73, $p\leqslant0.05$**<br>**9.66 km/h: 5.23, $p\leqslant0.05$**

11.28 km/h: 10.40, p≤0.05

Cohen's d

Wrist (left):

4.80 km/h: 1.58

6.42 km/h: 4.03

8.04 km/h: 9.74

9.66 km/h: 10.88

11.28 km/h: 11.54

Hip (right):

4.80 km/h: 0.36

6.42 km/h: 0.39

8.04 km/h: 0.36

9.66 km/h: 0.49

11.28 km/h: 1.08

Pearson's r

Wrist (left):

4.80 km/h: 0.105, (p>0.05)

6.42 km/h: 0.030, (p>0.05)

8.04 km/h: -0.169, (p>0.05)

9.66 km/h: -0.165, (p>0.05)

11.28 km/h: -0.130, (p>0.05)

Hip (right):

4.80 km/h: 0.529, p<0.001

6.42 km/h: 0.428, p<0.001

8.04 km/h: 0.110, (p>0.05)

9.66 km/h: 0.210, p<0.001

11.28 km/h: 0.162, (p>0.05)

	ICC<br>Wrist (left):<br>0.073<br>Hip (right):<br>0.868<br><br>MPE, % (calculated with group values)<br>Wrist (left):<br>4.80 km/h: -21,4<br>6.42 km/h: -41,4<br>8.04 km/h: -52,0<br>9.66 km/h: -58,1<br>11.28 km/h: -63,9<br><br>Hip (right):<br>4.80 km/h: -5,7

	6.42 km/h: -6,3<br>8.04 km/h: -4,7<br>9.66 km/h: -5,2<br>11.28 km/h: -10,4

Statistical significance was bolded. ICC: intraclass correlation; MAPE: mean absolute percentage of error; MPE: mean percentage of error.
