# Supplementary material for: Current Knowledge about ActiGraph GT9X Link Activity Monitor Accuracy and Validity in Measuring Steps and Energy Expenditure: A Systematic Review
Source: Sensors (Basel). 2024 Jan 26;24(3):825. doi: 10.3390/s24030825 (PMC10857518; doi:10.3390/s24030825)
Supplement: Supplementary file 1 [file sensors-24-00825-s001.zip › Supplementary Table S4.pdf]

Supplementary Table S4. Individual quality items of included studies with scores across reporting, external validity, and internal validity sub-scales.

		Toth et al., 2018 [19]	John et al., 2018 [36]	Ata et al., 2018 [34]	Tedesco et al., 2019 [39]	Ho et al., 2019 [35]	Lynn et al., 2020 [37]	Mora-Gonzalez et al., 2022 [38]	Anens et al., 2023 [33]
**Reporting**	Q1	1	1	1	1	1	1	1	1
	Q2	1	1	1	1	1	1	1	1
**(/9)**	Q3	1	0	1	1	1	1	1	1
	Q4	0	0	0	0	0	0	0	1
	Q5	1	1	1	1	1	1	1	1
	Q6	0	0	1	0	0	0	1	1
	Q7	1	1	1	0	1	1	1	1
	Q8	1	1	0	1	1	1	1	1
	Q9	1	1	1	1	0	0	1	1
	**Total**	**7**	**6**	**7**	**6**	**6**	**6**	**8**	**9**
**External Validity**	Q10	CD	CD	CD	CD	CD	CD	CD	CD
	Q11	0	0	0	0	0	0	0	0
**(/3)**	Q12	1	1	1	1	1	1	1	1
	**Total**	**1**	**1**	**1**	**1**	**1**	**1**	**1**	**1**
**Internal Validity**	Q13	0	0	0	0	0	0	0	0
	Q14	1	1	1	1	1	1	1	1
**(/7)**	Q15	1	1	1	1	1	1	1	1
	Q16	1	1	1	1	1	1	1	1
	Q17	1	1	1	1	1	1	1	1
	Q18	1	1	1	1	1	1	1	1
	Q19	0	0	0	0	0	0	0	0
	**Total**	**5**	**5**	**5**	**5**	**5**	**5**	**5**	**5**

CD: cannot determine
